# Mortality among individuals with substance use disorder—does violent criminal behavior have an impact?

**DOI:** 10.3389/fpsyt.2024.1455343

**Published:** 2024-12-06

**Authors:** Joakim Jakobsson, Anna Karlsson, Anders Håkansson, Björn Hofvander

**Affiliations:** ^1^ Lund Clinical Research on Externalizing and Developmental Psychopathology, Department of Clinical Sciences Lund, Lund University, Lund, Sweden; ^2^ Centre of Ethics, Law and Mental Health, Department of Psychiatry and Neurochemistry, University of Gothenburg, Gothenburg, Sweden; ^3^ Department of Forensic Psychiatry, Skåne University Hospital, Trelleborg, Sweden; ^4^ Department of Clinical Sciences Lund, Psychiatry, Faculty of Medicine, Lund University, Lund, Sweden

**Keywords:** violent offending, mortality, substance use disorder, psychiatry, somatic disease, preterm mortality

## Abstract

**Introduction:**

Understanding violent criminality and its impact on health and eventually the risk of premature mortality is important for efficient future interventions. This study aimed to explore the effect violent criminality had on premature mortality (i.e., death before the age of 65) among individuals with substance use disorders (SUDs).

**Methods:**

The cohort was created by identifying all Swedish patients diagnosed with SUD between the first of January 2013 and 31st of December 2014. The individuals were split into three age categories.

**Results:**

There were significant differences in standard mortality rates (SMR) in the cohort compared to the general Swedish population across the three age categories. We found differences between the SMRs for individuals convicted of violent and nonviolent crimes in the two younger age categories [age 15–29: violent crime (42.4) vs. non-violent crime (36.6), age 30–44: violent crime (28.0) vs. non-violent crime (23.0)]. A Cox regression analysis showed that each conviction of a violent crime increased the hazard ratio (HR) of premature mortality significantly [age 15–29; HR = 1.10 (95% CI: 1.04–1.17), age 30–44; HR =1.06 (95% CI: 1.03–1.09)]. After correcting for non-violent crimes, the increased risk only remained for the youngest group [HR = 1.06 (95% CI: 1.00–1.13)].

**Discussion:**

This study suggests that criminal behavior constitutes a proxy for the risk behaviors that increase the risk of premature mortality among young individuals with SUD even after controlling for confounders. Longitudinal studies, examining time-dependent risks and protective influences, are needed to explain the different pathways and processes leading to the amplified premature mortality in the groups.

## 1 Introduction

Abuse of both legal and illegal substances is an increasing problem in western society and is linked to a reduced life expectancy in several industrialized countries (1, 2). Additional to being an issue with socio-political implications, substance abuse and substance use disorders (SUDs) are related to suffering and adverse outcomes among the affected and their kin (3). Regarding legal substances, alcohol is known to cause many issues on both societal and individual levels (4). A meta-analysis on mortality among alcohol abusers, which included 81 observational studies and 853,722 individuals, showed a higher-than-expected relative risk (RR) for premature mortality (RR = 3.38) and that this risk is especially high among individuals under 30 years of age (RR = 9.42) and women (RR = 4.57).

The same issues and adverse outcomes are also associated with illegal substances. A large-cohort study comprised of individuals in treatment for illicit drug use between 1996 and 2006 was performed in Denmark (5), with a total of 111,445 person-years analyzed. Throughout the study, a total of 1,441 deaths were recorded, and many illicit substances were associated with severely increased standardized mortality rates (SMRs) compared to the general population. This included substances such as cannabis (SMR = 4.9), cocaine (SMR = 6.4), amphetamine (SMR = 6.0), heroin (SMR = 9.1), and other opioids (SMR = 7.7).

Besides premature mortality, a range of other adverse outcomes have been shown among people with SUDs; among these is an increased probability of violent behavior and legal problems (6). Although the causal chain is not completely clear, SUD is overrepresented in criminal and violent populations (7). The opposite relationship is likewise true; crime and violence are also overrepresented in the SUD population (8, 9). As an example, Pierce and colleagues (8) followed a group of individuals from drug naivety to initiation of opioid abuse. They found a severely increased probability of these individuals committing crime (RR 1.99 for men and RR 4.59 for women) after drug use onset. They also found that opioid initiation exacerbated this risk for many crime categories, with an overall increased relative risk ratio of 16% for men and 100% for women.

One theory that seeks to explain this is the Tripartite Model, proposed by Goldstein (10), which suggests that substance use disorder (SUD) is hypothetically linked to three distinct pathways leading to violent behavior. These pathways are absent in individuals without SUD (10, 11). In short, the first pathway is characterized by the effect that drugs have on cognition, leading to an intensified emotional state and violent behavior. The second describes the violent pathway due to the individuals’ interactions with drug distributors and/or other drug users. Finally, the third pathway is characterized by economic violence when financial difficulties arising from the acquisition of drugs result in incidents such as robberies.

The risk for premature death among offenders is well known, and mortality rates seem to be considerably higher compared to the general population (12). This association has been shown for several types of offenders: mentally ill offenders (13), incarcerated offenders (14), and offenders recently released from incarceration (15). Importantly, studies suggest that individuals exhibiting a violent behavior have a particularly high likelihood of premature mortality (16, 17). As mentioned, a criminal lifestyle is associated with other risk behaviors such as substance use and its conjoined risks (18). A Swedish study showed that the combination of violent behavior and an SUD diagnosis resulted in an almost fourfold higher hazard of premature mortality compared to their equals without SUD diagnoses (19). Among repeat violent offenders, a fourfold increased hazard rate for suicide was found, which decreased to about a twofold risk after controlling for inpatient psychiatric care. Therefore, there is reason to believe that the high mortality rates, at least partly, can be explained by psychiatric morbidity.

Among individuals with SUDs as well as the criminal population, there is a high prevalence of psychiatric disorders (15). This group is also more prone to risk behaviors associated with somatic illnesses such as smoking and bad eating habits (18, 20, 21), ultimately leading to a high probability of diabetes, cardiovascular disease, and premature mortality (22–24).

In a systematic review on premature death among offenders conducted by Skinner and Farrington (12), the authors call for further research that will examine the impact of offending while controlling for relevant risk factors. To the authors’ knowledge, there are a few studies on how violent offending affects outcomes of premature mortality after controlling for both SUDs and psychiatric morbidity (19, 25). Importantly, there is a knowledge gap concerning the interplay between SUD and violent criminal behavior in different age groups, controlling for other important risk factors. Based on previous research, we would expect synergistic effects of violent offenses and SUD on the propensity for early death, but this effect might be altered with age due to a number of moderators, among them are biological factors such as reaching cognitive maturity (26), the increased likelihood of desisting from crime with age (27), and the increased somatic morbidity and health issues among older offenders (28).

In this study, we aimed to describe the incidence of mortality in a large nationwide cohort of Swedish individuals with SUD and the incremental effects that violent offending might have on premature mortality, controlling for SUD and psychiatric morbidity.

The aims of the present study were to (1) describe premature mortality and causes of premature mortality in this population, (2) investigate whether violent criminality has an incremental effect on the risk for premature mortality, and lastly, (3) explore if this relationship persists in different age categories.

## 2 Methods

### 2.1 Participants

The cohort was established by identifying all inpatients and individuals who received specialized care, including both in- and outpatient psychiatric clinic services, who were diagnosed with substance use disorder (ICD-10 codes F10.0–F19.9) in Sweden between January 1, 2013 and December 31, 2014. The exclusion of retrospective diagnostic data was intended to maximize the probability of a cohort consisting of individuals with active substance use disorder in the current healthcare landscape. Data was collected through the Swedish National Patient Register (NPR), held by the Swedish National Board of Health and Welfare. The register contains Swedish healthcare data from inpatient and specialized healthcare visits and has high validity regarding diagnosis (29). The current study includes individuals from the age of 18 until 65 years at the end of the study.

Data were merged and anonymized by an independent government agency (30), and the code linking the personal identification numbers to the new case of numbers was destroyed immediately after merging. Data were accessed for research purposes on the 21st of February 2021.

### 2.2 Psychiatric morbidity and SUD

Data on morbidity was collected from the NPR, which is reported to be valid and dependable for a range of psychiatric diagnoses (29). The data consists of all diagnoses received, through ICD 10 codes, in all inpatient and specialized care events during follow-up. Thus, each individual could have received multiple diagnosis during follow-up. We categorized psychiatric morbidities as follows: psychotic disorder (F20-F29), bipolar disorder (31.0-F31.9), depression (F32.0-F33.9), anxiety (F41.0-F41.9), antisocial personality disorder (F60.2), other personality disorder (F60.0-F60.1, F60.3-F69.9), and ADHD (F90.0B). Specific SUDs were categorized as opioid (F11.0-F11.9), cannabis (F12.0-F12.9), sedatives (F13.0-F13.9), central stimulants (F14.0-F15.9), alcohol (F10.0-F10.9), and multiple drug use (F19.0-F19.9).

### 2.3 Criminal history

Complete official register-based criminal history was collected from the Crime Register, held by the National Council of Crime Prevention. The register dates to the first of January 1973 and holds information on all convictions in the Swedish lower courts regarding individuals aged 15 years and up. The included patients’ conviction data was collected from 18 years of age until the end of follow-up, 31st of December 2017. The offenses are defined in the Swedish Penal Code (45:700), Narcotic Drugs Punishment Act (46:64), and the law on punishment of certain traffic offenses (47:649), including court convictions, order of summary punishment, and omission of prosecution. The register has been shown through external validation to have a high accuracy concerning convictions for violent crime (31). In this paper, we used the number of court convictions, violent or non-violent, which could include several offenses each. We split the convictions into categories of non-violent and violent where violent crime is defined by the National Council of Crime Prevention (32), which classifies crimes against a person as a violent crime. This includes the crimes of murder, manslaughter, assault, robbery, rape, and/or violence against an officer. A total of 139 individuals had been punished for a violent crime, without a court conviction in a process called abstention from prosecution. This means that while the crime is investigated, there is no formal indictment or trial. However, the waiver has the same legal effect as a conviction. There are several reasons for an abstention from prosecution, such as the individual is under the age of 18, the individual has been convicted of another crime and a new offense would not lead to a harsher penalty, or if the crime is unlikely to result in a more severe punishment than a fine or conditional sentence. These individuals were categorized as violent offenders.

### 2.4 Mortality

Data on mortality was collected from The Swedish Cause of Death register (CDR). The register contains data on all deaths of people registered in Sweden and is maintained by the Swedish National Board of Health and Welfare. Swedish physicians are obliged to report these data to the National Board of Health and Welfare within 3 weeks from the time of death (33).

The included patients’ mortality data was collected from inclusion until the end of follow-up, 31st of December 2017. All deaths due to homicide must be reported by the physician performing the forensic autopsy, and 95% of the deaths due to suicide are reported as well. Deaths among individuals with known or suspected alcohol or drug use disorders are also routinely investigated through forensic autopsy (33). In 90% of forensic autopsies in Sweden, a comprehensive analysis of alcohol, pharmaceuticals, and illicit drugs is performed (33).

The quality of the Swedish CDR has been estimated through comparison to case reports looking at individuals who passed away in hospitals, and 77% agreement on an underlying cause of death was found (34). The accuracy was particularly high regarding the groups studied in this paper (age 0–44: 98%, age 45–64: 91%) (34).

In this current study, the information on causes of death is described in accordance with ICD-10. The causes of death are categorized as follows: somatic death (A00-R99), accidental fatal intoxication (X40-49), assault (X85-Y09), suicide (X60-X84), and accidents (W00-W99). We chose not to include diagnoses classified as “undetermined event” (i.e., ICD-10 codes X40–X49 and/or Y10–Y34) in the suicide or self-harm categories but to keep these apart as suggested by Björkenstam and colleagues (34).

### 2.5 Statistical analysis

SMRs, including confidence intervals, were calculated through comparison with the Swedish population. To assess the prevalence of causes of death during the follow-up period, odds ratios (ORs) were calculated for each cause of mortality as a binary event at the conclusion of the study. This was done using univariate logistic regression, with non-convicted individuals in the cohort serving as the reference group. The SMRs describe overall mortality events, stratified by violent crimes, at different age categories, per 100,000 individuals: 15–29, 30–44, and 45–64 years of age for all patients. Data on mortality during the follow-up time for the general Swedish population were retrieved from an open access service on Statistics Sweden’s official website (30). The data provided by Statistics Sweden is displayed as number of deaths per 100,000, stratified per age group.

For within-group data, *t*-tests were used for continuous variables and chi-square test was used for binary as well as categorical data. All associations lower than *p <*0.30 were included in the Cox regression analysis. The proportional hazard assumption limit was set to >*p* = 0.05. Both simple and multiple-factor Cox regression analyses were conducted to investigate known and hypothesized risk factors of premature mortality. The results were presented as hazard ratios (HR) and described with 95% confidence interval. All data were analyzed using the STATA 17 statistical software. The level of statistical significance was set to 0.05.

### 2.6 Ethics approval and consent to participate

The study was approved by the regional ethics committee of Lund, Sweden (file number: 2018/3). The study procedures were conducted in accordance with the Declaration of Helsinki. The ethics committee of Lund waived the need for informed consent as the study was based on administrative population-based registers.

## 3 Results

### 3.1 Clinical characteristics

The patients (*N* = 90,181) were followed for an average of 1,449 days (3.9 years) and those deceased before the study’s end for an average of 674 days (1.8 years).

As shown in **Table 1**, the type of substances used differed between the age groups. Cannabis use was more common in the youngest age group (26.6%), while opioid use stood out among the middle age group (22.4%). Several SUDs were associated with premature mortality among the two younger age groups but not the oldest one (opioids, multiple drug use), while cocaine use was only associated with premature mortality among 30–44-year-olds. The highest mean number of violent crimes was found among the group aged 30-44 at the study’s end.

**Table 1:** Baseline characteristics by age group and binary associations with premature mortality divided by age group.

Age group	15–29 years *n* = 22 833 (SD, %)	30–44 years *n* = 24 762 (SD, %)	45–64 years *n* = 42 586 (SD, %)
Age, study end (mean)	24.6 (3.1)	36.6 (4.4)	55.1 (5.5)
Follow-up time, days (mean)	1,442 (293)	1,485 (315)	1,432 (393)
Number convicted, all crime	6,812 (29,8) ***	10,754 (56.4) ***	12,030 (28.2)
Violent crime	3,434 (15.0) ***	6,345 (25.6)	6,702 (15.7) *
Psychotic disorders	1,496 (6.6) ***	2,571 (10.4) *	2,745 (6.4)
Bipolar disease	1,281 (5.6) *	2,114 (8.5)	2,734 (6.4) ***
Depression	5,192 (22.7) ***	6,430 (26.0) ***	9,080 (21.3) ***
Recurrent depression^a^	1,715 (7.5) *	2,762 (11.1) ***	4,129 (9.7) ***
Anxiety	6,961 (30.0) ***	8,617 (34.8) **	10,129 (23.8) ***
Other personality disorders	2,394 (11.3) ***	3,432 (16.0)	2,746 (7.2) ***
Antisocial personality disorder	193 (0.8) ***	548 (2.2) **	304 (0.7) **
ADHD	4,421 (19.3) ***	4,837 (19.5)	3,410 (8.0) ***
Opioids	2,323 (10.1) ***	5,557 (22.4) ***	5,022 (11.8) *
Cannabis	6,080 (26.6) ***	3,179 (12.8) ***	1,319 (3.1) ***
Sedatives	2,611 (11.4) ***	4,459 (18.0) ***	4,473 (10.5) ***
Cocaine	389 (1.7)	551 (2.2) ***	234 (0.6)
Other central stimulants	1,650 (7.2) ***	2,854 (11.5) ***	2,441 (5.7) ***
Multiple drug use	7,844 (34.3) ***	10,490 (42.3) ***	8,302 (19.5) *
Alcohol	12,652 (55.4) ***	13,090 (52.9) ***	28,874 (67.8) ***

a More than two episodes.

*p < 0.30, **p < 0.05, ***p < 0.01.

### 3.2 Standardized mortality rates

The SMRs for overall mortality were calculated for each category depending on age and violent crime status (presented in **Table 2**). While all groups had a significantly increased risk of premature death compared to the general population from previous literature, the highest SMRs were found in the youngest age group (30). In the age categories, the 15–29 and 30–44 groups with violent convictions had the highest SMRs, and groups with other convictions had the second highest. This clear distinction could not be found in the oldest age category, 45–64 years of age, where the CIs are overlapping (no conviction vs. violent conviction).

**Table 2:** SMRs calculated from all-cause mortality stratified by convictions and age.

All-cause mortality	No sentence		Sentenced for non-violent crime		Sentenced for violent crime		Total	
	SMR	95% CI	SMR	95% CI	SMR	95% CI	SMR	95% CI
Age 15–29	13.4	12.3**–**14.5	36.6	35.0**–**38.6	42.4	40.5–44.4	21.3	19.9–22.7
Age 30–44	14.0	13.2**–**14.9	23	21.9**–**24.1	28.0	26.8–29.2	19.3	18.3–20.3
Age 45–64	8.7	8.4**–**9.0	7.8	7.5**–**8.1	8.6	8.3–8.9	8.5	8.2–8.9

### 3.3 Causes of death

During the study period, a total of 7,141 patients (7.9%) passed away. The leading causes of death differed between age categories, but intoxication and suicide were common in all groups. Death through a somatic disease became more probable with age and was the leading cause of death in the oldest age category. There was an increased risk for several causes of death compared to the reference population of individuals with no convictions in the cohort. In the two younger age groups, a very high OR for death by assault was found (OR = 23.26 for patients aged 15–29 and OR = 8.84 for patients aged 30–44). A full list of causes of deaths, divided into age categories, can be found in **Tables 3.1**–**3.3**.

**Table 3.1:** Causes of death among individuals between 15 and 29 divided by type of conviction.

	No conviction *n* = 16,000		Nonviolent conviction *n* = 3,399		Violent conviction *n* = 3,434	
Causes of death	*n* (%)	OR	*n* (%)	OR	*n* (%)	OR
Somatic disease	35 (10.1)	1.00	18 (9.8)	2.44 (1.38–4.31)	18 (7.5)	2.40 (1.36–4.45) **
Fall	3 (0.1)	1.00	0 (0.00)	–	4 (1.7)	6.22 (1.39–27.80) *
Accident	11 (3.1)	1.00	8 (3.9)	3.43 (1.38–8.53) **	6 (2.5)	2.54 (0.94–6.88)
Accidental poisoning	113 (32.6)	1.00	107 (52.2)	4.57 (3.50–5.97) **	114 (47.7)	4.82 (3.71–6.28) **
Intentional self-harm	118 (34.0)	1.00	30 (14.6)	1.20 (0.80–1.79)	48 (20.1)	1.91 (1.36–2.67) **
Undetermined cause	65 (18.7)	1.00	35 (17.1)	2.55 (1.69–3.85) **	39 (16.3)	2.82 (1.89–4.20) **
Hospital complications	0 (0.00)	–	0 (0.00)	–	0 (0.00)	–
Assault	2 (0.6)	1.00	7 (3.4)	16.51 (3.43–79.50) **	10 (4.2)	23.36 (5.12–106.67) **
Total	347 (100)	1.00	205 (100)	2.90 (2.42–3.46) **	239 (100)	3.37 (2.85–4.00) **

*p < 0.05, **p < 0.01.

**Table 3.2:** Causes of death classified among individuals between 30 and 44 divided by type of conviction.

	No conviction *n* = 13,986		Nonviolent conviction *n* = 4,431		Violent conviction *n* = 6,345	
Causes of death	*n* (%)	OR	*n* (%)	OR	*n* (%)	OR
Somatic disease	176 (31.7)	1.00	49 (16.6)	0.88 (0.64–1.21)	107 (20.9)	1.35 (1.06–1.72) *
Fall	14 (2.7)	1.00	6 (2.1)	1.35 (0.52–3.52)	11 (2.5)	1.73 (0.79–3.82)
Accident	6 (1.1)	1.00	4 (1.4)	2.11 (0.59–7.47)	15 (2.9)	5.52 (2.14–14.24) **
Accidental poisoning	119 (21.4)	1.00	144 (48.7)	3.91 (3.06–5.00) **	228 (44.5)	4.34 (3.47–5.43) **
Intentional self-harm	147 (26.5)	1.00	45 (15.2)	0.97 (0.69–1.35)	54 (10.5)	0.80 (0.59–1.11)
Undetermined cause	87 (15.7)	1.00	44 (14.9)	1.60 (1.11–2.31) *	81 (15.8)	2.07 (1.52–2.80) **
Hospital complications	2 (0.4)	1.00	0 (0.00)	–	0 (0.00)	–
Assault	4 (0.7)	1.00	4 (1.4)	3.16 (0.79–12.63)	16 (3.1)	8.84 (2.95–26.44) **
Total	555 (100)	1.00	296 (100)	1.73 (1.50–2.00) **	512 (100)	2.12 (1.88–2.40) **

*p < 0.05, **p < 0.01.

**Table 3.3:** Causes of death among individuals between 45 and 64 divided by type of conviction.

	No conviction *n* = 30,531		Nonviolent conviction *n* = 5,353		Violent conviction *n* = 6,702	
Causes of death	*n* (%)	OR	*n* (%)	OR	*n* (%)	OR
Somatic disease	2 851 (79.7)	1.00	379 (63.8)	0.74 (0.66–0.83) **	516 (63.2)	0.81 (0.74–0.89) **
Fall	99 (2.7)	1.00	22 (3.7)	1.22 (0.80–2.02)	27 (3.3)	1.24 (0.81–1.91)
Accident	37 (1.0)	1.00	19 (3.1)	2.94 (1.69–5.11) **	18 (2.2)	2.22 (1.26–3.90) **
Accidental poisoning	184 (5.1)	1.00	70 (11.8)	2.19 (1.66–2.88) **	148 (18.1)	3.72 (3.00–4.63) **
Intentional self-harm	232 (6.5)	1.00	49 (8.2)	1.21 (0.89–1.64)	52 (6.4)	1.02 (0.76–1.38)
Undetermined cause	156 (4.4)	1.00	54 (9.1)	1.98 (1.45–2.71) **	48 (5.8)	1.41 (1.02–1.94) *
Hospital complications	8 (0.2)	1.00	0 (0.0)	–	3 (0.4)	1.71 (0.45–6.44)
Assault	10 (0.3)	1.00	1 (0.2)	0.57 (0.07–4.46)	4 (0.5)	1.82 (0.57–5.81)
Total	3 577 (100)	1.00	594 (100)	0.94 (0.86–1.03)	816 (100)	1.05 (0.96–1.13)

*p < 0.05, **p < 0.01.

### 3.4 Predicting premature death

Cox proportional hazard regressions were performed to relate several known risk factors to survival time. The proportional hazard assumption was met for total violent crimes in two age groups. However, for many of the controlling variables (age 15–29: antisocial personality disorder, alcohol, cannabis, sedatives, opioids, and multiple drug use; age 30–44: cannabis, sedatives, opioids, multiple drug use, and central stimulants), the proportional hazard assumption could not be met due to a lack of proportionality over time. The HRs presented for these variables should therefore be considered an average effect over time. The variable depression was excluded from the final models due to its opposing effect direction over time. In **Tables 4.1**, the Cox models showed that the number of convictions for violent crime significantly increased the risk of premature mortality among age groups 15–29 and 30–44 by approximately 10% and 6%, respectively, for each additional conviction. This correlation remained borderline significant after controlling for several established risk factors for premature mortality among substance users. However, as can be seen in **Tables 4.2**, after controlling for non-violent crimes, the result was no longer significant (*p* = 0.05) among 30–44-year-olds.

**Table 4.1:** Independent, age group-dependent, and Cox regressions models predicting mortality.

Variables	HR (95% CI)	
	Age 15–29	Age 30–44
Total violent crimes	1.10 (1.04–1.17) **	1.06 (1.03–1.09) **
Male	2.20 (1.84–2.62) **	1.45 (1.27–1.66) **
Other personality disorders	1.42 (1.17–1.74) **	–
Alcohol	0.73 (0.63–0.85) **	–
Cannabis	0.59 (0.49–0.70) **	0.56 (0.48–0.67) **
Sedatives	1.71 (1.45–2.02) **	1.29 (1.13–1.47) **
Opioids	1.55 (1.31–1.84) **	1.33 (1.18–1.50) **
Multiple drug use	3.61 (3.02–4.31) **	2.09 (1.85–2.36) **
Cocaine	–	0.40 (0.24–0.66) **

*p < 0.05, **p < 0.01.

**Table 4.2:** Independent, age group-dependent, and Cox regressions models predicting mortality, including non-violent crimes.

Variables	HR (95% CI)	
	Age 15–29	Age 30–44
Total violent crimes	1.06 (1.00–1.13) *	1.03 (1.00–1.06)
Crimes, non-violent	1.00 (0.96–1.03)	1.02 (1.00–1.03) *
Male	2.30 (1.92–2.75) **	1.46 (1.27–1.66) **
Other personality disorders	1.53 (1.25–1.87) **	–
Alcohol	0.73 (0.63–0.85) **	–
Cannabis	0.55 (0.46–0.55) **	0.57 (0.47–0.69) **
Sedatives	1.74 (1.47–2.06) **	1.27 (1.11–1.44) **
Opioids	1.62 (1.36–1.93) **	1.29 (1.14–1.46) **
Multiple drug use	3.98 (3.31–4.79) **	2.11 (1.86–2.38) **
Cocaine	–	0.40 (0.24–0.67) **

*p < 0.05, **p < 0.01.

## 4 Discussion

This current paper studied the incremental effect violent offending might have on premature mortality among a cohort of patients suffering from SUD. Previous studies have shown an increased risk of premature mortality among both offenders and substance users, but few studies have controlled for SUD and psychiatric morbidity to study violent criminality as a moderator on premature mortality. Little was also known about how and whether a violent crime plays a role for premature mortality in different age groups. In this study, we split our data into three age groups: 15–29, 30–44, and 45–64 years at the study’s end. We did this for two reasons: because it follows the current cutoff ages for data on premature death from the Swedish public health authority and it follows the established propensity of crime trajectory (27).

Our first aim was to describe the mortality within the cohort. We could show that all patients with SUD had much higher SMRs compared to the general Swedish population based on national statistics (30). In addition, the SMRs among SUD patients were also higher than previously shown in previous literature (5) but comparable to the high mortality rates among persistent offenders (35). We believe this to be a combination of the synergistic effects of criminal activity and the high risk of an active SUD in the cohort and the very low number of deaths per 100,000 inhabitants in Sweden during the follow-up (36). In recent years, the “opioid crisis” has become a well-known term due to its strong effects on premature mortality. Drug overdoses, mostly caused by opioids and opiates, are now the number one cause of death among people under the age of 50 in America (37, 38). While not as evident, some research suggests that a similar trajectory can be found in European countries (39). In our results, a high percentage of the early deaths, especially among the two younger age groups (age 18–29, 42.2%; age 30–44, 36.0%; age 45–64, 8.1%), were related to accidental poisoning, which could mirror similar conditions. When analyzing the within-group data, it is important to remember that the cohort exists exclusively of patients with SUDs. Our Cox regression analysis showed a decreased risk of premature mortality for several SUDs (alcohol and cannabis) among patients aged 18–44 but also that opioid use and multiple drug use severely increased the risk. However, opioid use and multiple drug use could not solely explain the difference between the groups of violent and non-violent individuals in our cohort.

Our second and third aim sought to investigate whether violent criminality had an incremental effect on premature mortality and whether this varied by age. While all groups had a severely increased risk for premature mortality, the individuals that really stood out were the ones sentenced for at least one violent crime. As shown in **Table 2**, in line with our hypothesis, we found the SMRs for violent individuals to be sharply elevated but decreasing with age. Although the increased risk of premature mortality among individuals with SUD and criminal convictions is well known, the amplitude in our study was surprising. A Finnish study of general offenders showed a linear association between the number of conviction and the risk of premature mortality (35). Highly persistent offenders (convicted for 28 or more crimes) showed ORs comparable to ours, while less crime-prone offenders showed a much more modest risk figure. A Swedish study, similar to ours, though researching mortality in criminal justice clients with substance use problems, showed considerably lower SMRs (Women; 7.0, Men 7.7) (40). However, none of the above and no research, to the authors’ knowledge, studied mortality among offenders by type of conviction.

The causes underlying the differing mortality rates between individuals convicted of violent and non-violent crimes and those never convicted are undoubtedly complex and multifaceted. Numerous studies have shown a severely increased risk of dying through unnatural causes after the release from prison, especially among offenders with SUD (14, 15, 41). This has been studied in a Swedish setting, where a follow-up study researching unnatural death among Swedish offenders showed that the risk of premature mortality was particularly increased for individuals who had SUD (42).

As predicted and shown by the HRs as well as the SMRs, the effect of violent crime convictions on premature mortality decreased with age. For individuals 18–29 years of age, this risk was enhanced by 11% for each violent conviction, while the respective number for individuals aged 30–44 was 6%, even after testing for known confounders such as drug use and psychiatric morbidity. However, when including non-violent convictions to the Cox regression analysis, the additional risk for each violent conviction was limited to 6% for patients 18–29 years old and did not remain in the older age group.

While the act of violent crime can constitute a risk of severe somatic consequences, we believe that violent criminality works better as a proxy for other risk behaviors. Therefore, we believe that the age-dependent decrease is, at least partly, due to two well-documented phenomena: the sharply deteriorated somatic health among former offenders and older substance abusers and the age-dependent decrease in criminal activity. Previous studies have highlighted the deteriorating health effects of being an offender and/or substance abuser (43, 44). In many cases, death through somatic causes have been preceded by health issues with symptoms limiting activity. This could, in turn, lead to a lifestyle with less risk-taking, thus limiting the behavioral differences of offenders compared to the general population and consequently leading to less excessive unnatural deaths compared to the younger age groups. Our results displaying causes of death also provide some pieces of evidence in line with this assumption. We could show that deaths through accidental intoxication and violence were more common among younger individuals. Death through a somatic disease, on the other hand, only accounted for 9% of all mortality in the youngest age group compared to the oldest age group where somatic disease accounted for more than 75% of all premature deaths. This mirrors the causes of deaths in different age groups in the general population where unnatural deaths such as suicide and intoxication are more probable at a younger age while death through a somatic disease becomes more probable with age (36). Interestingly, convictions decreased the probability of dying through somatic disease in the oldest age group. We believe that this is due to the physical nature of a criminal lifestyle, e.g., a certain level of somatic health is needed to commit a crime, thus skewing the group division. However, more studies are needed for adequate interpretation.

Regarding the age-dependent decrease in criminal activity, the age–crime curve shows that the prevalence of offending behavior reaches its lifetime peak somewhere between middle/late adolescence and up to young adulthood. Thereafter, it decreases until elderdom. Though we do not know when our cohort committed the crimes, previous data suggests this to be a common trajectory among offenders. The famous trajectory is one of the most consistent findings within criminology and has been replicated both over time and location (27, 45). Therefore, it is less likely for the older patients to be criminally active and experiencing adverse life events connected to a lifestyle of crime, drug use, and prison sentences (19). Currently, interventions reducing the mortality rates among offenders have been largely unsuccessful and even increased the risk of unnatural death in some cohorts (46). However, due to the pattern of death causes, there is a belief that interventions regarding drug use, suicide prevention, and crime reduction might be successful in limiting premature mortality. Consequently, future studies researching age-specific risk factors, effective interventions, and individual turning points are needed to explain the effect that violent behavior has on premature mortality and how to prevent it.

Our study has seven main limitations: (1) our cohort is based solely on individuals getting an SUD diagnosis in a specialized healthcare setting and thus does not include drug-addicted individuals without contact with the Swedish healthcare system. Moreover, individuals with lesser substance use problems, who are being handled in primary care, are not included. The results are therefore not generalizable to them. (2) The lifetime prevalence of convictions as well as SUD diagnosis varied considerably between the two younger and the oldest age group. Due to the cross-sectional approach of inclusion, it is plausible that the older age group differed in other aspects not accounted for in this study. (3) We did not have data on injection drug use, which is a factor known to severely increase the risk of unnatural death (47). (4) We did not have access to time-dependent data on criminal convictions. As a result, we were unable to distinguish between self-limiting, persistent, past, or more recent violent behavior, which likely exerts a varying impact on the probability of premature mortality within each age group (48). (5) We could not control for somatic morbidity. Closely associated with premature mortality and probably pejorative in relation to physical activation, somatic morbidity would have been important to include. (6) Information on crime and mortality among patients moving abroad after inclusion is missing and could have influenced the results. (7) No data on sociodemographic or familial factors were available for this study; inclusion of this data might have influenced the results as confounders.

To conclude, our study suggests an additive effect of each violent offense, operationalized as convictions on premature mortality among individuals with SUDs between 18 to 29 years of age. This effect withstood correcting for all SUDs, psychiatric morbidity, and several known predictors of premature mortality. While further studies will be necessary to establish the causal links between SUD, violence, and premature mortality, we believe that this is an important step to future interventions and successful identification of high-risk individuals.

## Data Availability

The raw data supporting the conclusions of this article will be made available by the authors, without undue reservation.
